# The Elastic Electronic Health Record: A Five-Tiered Framework for Applying Artificial Intelligence to Electronic Health Record Maintenance, Configuration, and Use

**DOI:** 10.2196/66741

**Published:** 2025-05-09

**Authors:** Colby Uptegraft, Kameron Collin Black, Jonathan Gale, Andrew Marshall, Shuhan He

**Affiliations:** 1 Technology Management & Integration Branch, Health Informatics Division, Defense Health Agency, Falls Church, VA, United States; 2 Department of Medicine, Stanford University School of Medicine, 291 Campus Drive, Stanford, CA, 94305, United States, 1 (650) 723-2300; 3 Department of Pediatrics, University of Minnesota, Minneapolis, MN, United States; 4 Department of Emergency Medicine, Harvard Medical School, Boston, MA, United States; 5 Department of Emergency Medicine, Massachusetts General Hospital, Boston, MA, United States

**Keywords:** semi-autonomous database, back-end EHR, self-configuring database, machine learning, health care, generative artificial intelligence, elastic EHR, electronic record, electronic health record, artificial intelligence, AI, EHR, database

## Abstract

Properly configuring modern electronic health records (EHRs) has become increasingly challenging for human operators, failing to fully meet the efficiency and cost-saving potential seen with the digitization of other sectors. The integration of artificial intelligence (AI) offers a promising solution, particularly through a comprehensive governance approach that moves beyond front-end enhancements such as user- and patient-facing copilots. These copilots, although useful, are limited by the underlying EHR configuration, leading to inefficiencies and high maintenance costs. To address this, we propose the concept of an “Elastic EHR,” which proactively suggests and validates optimal content and configuration changes, significantly reducing governance costs and enhancing user experience, as well as reducing many of the common frustrations including the documentation burden, alert fatigue, system responsiveness, outdated content, and unintuitive design. Our five-tiered model details a structured approach to AI integration within EHRs. Tier I focuses on autonomous database reconfiguration, akin to Oracle Autonomous Database functionalities, to ensure continuous system improvements without direct edits to the production environment. Tier II empowers EHR clients to shape system performance according to predefined strategies and standards, ensuring coordinated and efficient EHR solution builds. Tier III optimizes EHR choice architecture by analyzing user behaviors and suggesting content and configuration changes that minimize clicks and keystrokes, thereby enhancing workflow efficiency. Tier IV maintains the currency of EHR clinical content and decision support by linking content and configuration to updated guidelines and literature, ensuring the EHR remains evidence-based and compliant with evolving standards. Finally, Tier V incorporates context-dependent AI copilots to enhance care efficiency, quality, and user experience. Despite the potential benefits, major limitations exist. The market dominance of a few major EHR vendors—Epic Systems, Oracle Health, and MEDITECH—poses a challenge, as any enhancements require their cooperation and financial motivation. Furthermore, the diverse and complex nature of health care environments demands a flexible yet robust AI system that can adapt to various institutional needs that has not yet been developed, researched, or tested. The Elastic EHR model proposes a five-tiered framework for optimizing EHR systems and user experience with AI. By overcoming the identified limitations through vendor-led, collaborative efforts, AI-enabled EHRs could improve the efficiency, quality, and user experience of health care delivery, fully delivering on the promises of digitization within health care.

## Introduction

Properly and proactively configuring modern electronic health records (EHRs) has grown beyond human capabilities. As they are infinitely configurable, embedded potential and capabilities exist; however, properly configuring these capabilities at scale in a timely manner in an increasingly resource-constrained environment is not possible through the manual approaches of today. Fully leveraging this potential will require artificial intelligence (AI)-powered governance. AI integration with EHRs, however, has almost exclusively focused on front-end, user-, and patient-facing “copilots.” These copilots enhance navigating, searching, understanding, synthesizing, or documenting medical information. AI copilots have benefits, but they operate on a manually maintained, costly, and continuously noncurrent EHR content and configurations, ie, their effectiveness is fundamentally limited by flaws in the underlying EHR architecture. These flaws result from the complexity and scale of configurable “solutions” that comprise health record platforms; to solve this issue, we propose the “Elastic EHR”. We define this as an EHR that can proactively suggest and, upon validation, perform optimal configuration changes, significantly reducing governance costs and providing better user and patient experience. To specify the areas in which AI should target EHRs, we propose a five-tiered model (please see Table 1), each tier building upon the previous, with an emphasis on Tiers II-IV, as Tiers I and V have already been unofficially defined.

**Table 1.** Five tiers of an Elastic electronic health record (EHR).

Level	Tier	Description	Example
Users	Tier V: Copilots and Assistants	Context-dependent functionality designed to enhance care quality, efficiency, or experience for health care professions or patients.	A voice-enabled “copilot” assists clinicians during encounters, suggesting relevant diagnoses, auto-generating draft documentation, and proposed orders.
Configuration	Tier IV: External Knowledge Linkage	Suggested configuration changes based on evolving external evidence, autonomously executed, and communicated upon approval.	After the USPSTF^a^ updates its mammogram screening guidelines to start at age 40 years, Tier IV detects this change and proposes new EHR orders, forms, and documentation templates to ensure the organization’s screening recommendations and registries match the updated guidelines.
	Tier III: Workflow Optimization	Suggested configuration changes based on user behavior, autonomously executed, and communicated upon approval.	Tier III identifies a subset of clinicians who complete clinic visits more efficiently by using personal order sets, then merges these best-practice sets into a single enterprise-level order set for all physicians in that specialty, automatically queuing it for approval and release.
	Tier II: Internal Configuration Optimization	Suggested configuration changes based on client- and vendor-defined standards and the intended interactions between platform solutions.	An architect updates a patient intake form. Tier II suggests edits to maintain uniform naming conventions, default field values, and interface compatibility. It also highlights downstream solutions (eg, registries, templates) that might be impacted by any change.
Database	Tier I: Autonomous Database Tuning	Automated tuning, patching, and workload balancing, logged for administrator review.	Tier I automatically adjusts database indexes and memory allocations to optimize performance, creating a change log that flags issues such as slow queries or capacity constraints for subsequent human review.

a USPSTF: United States Preventive Services Task Force.

## Tier I: Autonomous Database Tuning

Tier I consists of autonomous database reconfiguration, operating similarly to the Oracle Autonomous Database with automated tuning, patching, and workload balancing [1]. This tier creates a change log for retroactive review, with examples including component upgrades, system maintenance suggestions, software error detection, cyber security threat detection, and supplemental database backups. To clarify, Tier I does not involve independent editing of the EHR production environment or any create, update, or delete functions.

## Tier II: Internal Configuration Optimization

### Introduction to Tier II

In Tier II, EHR clients shape the performance based on the desired “solution” strategy, style guides, and standardizations via approval and scheduling of recommended changes. Solutions are defined as a discrete set of functionality, including templated notes, auto text shortcuts (aka “dot phrases”), orders, order sets, and alerts. This tier optimizes connected EHR solution builds. For example, the Oracle Health EHR (formerly known as Cerner Millennium) contains up to 850 content and configuration tools, each with dozens to hundreds of options and subtools. The output of each of these tools may be connected to one or multiple other solutions. To build a simple form, we may require up to 12 distinct tools and a week of skilled architect time. The proper front-end flow depends on the coordinated build of solutions, but the tools to configure these solutions are siloed, and the solution architect or informaticist may be blind to the full system impacts. Changing the content of this form may negatively impact multiple other solutions, including note templates, orders, interfaces, and discrete data capture. Tier II ensures this hypothetical form is built both to institution-set standards and aligns with these other solutions.

### Tier II Scenario

A solution architect needs to update a form, a context-dependent collection of discrete data entry fields. However, multiple other solutions may populate or use these data fields, including results or data review solutions, “smart” documentation or ordering templates, outbound interfaces, logical rules or alerts, or patient registries. Without querying the database or system configurations for each potential impact, the architect is largely unaware of possible downstream ramifications. Tier II addresses this by providing AI-assisted guidance on how these solutions interact, automatically suggesting any necessary edits to keep interrelated components synchronized. In this process, a middleware layer becomes invaluable; it orchestrates data exchange among siloed EHR modules, allowing the AI engine to integrate seamlessly with the relevant system components. By maintaining consistent data structures and communication channels, middleware ensures that the architect’s updates are executed safely and comprehensively. Technically, this would include AI-generated queries of relevant database tables and system files, as well as a graphical user interface overlay that helps the architect visualize potential impacts and either approve or deny suggested changes.

### Evaluating Tier II

Tier II is designed to streamline how institutions sustain their EHRs, according to enterprise standards, while reducing configuration silos and ensuring that changes to one component do not inadvertently disrupt others. Evaluation metrics may include configuration turnaround time, error rates, compliance with institutional standards, and architect or administrator feedback*.*

#### Configuration Turnaround Time

This is the time to implement a specific EHR change—from the initial request to the final deployment. An effective Tier II system should significantly shorten this resolution process.

#### Error Rates

This is to monitor the frequency of errors or backouts after initial release. Fewer postdeployment fixes indicate that AI-driven guidance is proactively catching conflicts and dependencies.

#### Compliance with Institutional Standards

This is the alignment of new solutions with established style guides, templates, and regulatory requirements. A high compliance rate suggests that Tier II is helping maintain standardized, high-quality configurations.

#### Architect or Administrator Feedback

This includes qualitative feedback from solution architects, informaticists, and administrators about the system’s ease of use, clarity of recommended changes, and impact on daily workflows.

## Tier III: Workflow Optimization

### Introduction to Tier III

Tier III proactively suggests configuration changes to optimize EHR choice architecture. Optimal choice architecture in this context entails a configurable design that incentivizes the minimum number of clicks, keystrokes, and mouse miles to achieve an intended, quality outcome. Optimal architecture makes the right, efficient choice path the intuitive option, enhancing EHR usability [2]. With the current complexity of EHR design to account for the high variability within patient care, following the most efficient choice paths is not easy or intuitive. By globally monitoring user behavior and determining pockets of efficient users achieving defined process or outcome metrics, Tier III finds the ideal choice paths and suggests configuration changes to democratize them across all relevant user populations. It makes the easy path, the right path. Examples include identifying missing orders or default selections within order sets; optimizing note template content to reduce the manual insertion of discretely captured information or the unnecessary use of free text; updating default naming conventions and selections to reduce the misrouting of orders, notes, or messages; and consolidating unnecessary user positions, preferences, or roles.

### Tier III Scenario

An example scenario involves monitoring the ordering patterns of outpatient primary care physicians treating acute nasopharyngitis (common cold). Across hundreds of outpatient clinics, ordering times for these encounters vary widely, despite similar order volumes and medication classes. Tier III AI identifies a subset of providers who achieve faster, more efficient workflows by using personal order sets. The system consolidates these personal sets into a recommended, enterprise-level order set and queues its integration into the primary care physician’s workflow position. Once approved, the AI executes the change. To achieve this level of real-time monitoring and seamless deployment, a robust middleware solution can mediate data traffic, collecting operational metrics from disparate EHR modules, and pushing approved configuration changes into production.

### Evaluating Tier III

Tier III aims to optimize user workflows by identifying and disseminating best practices across relevant roles and settings. Key metrics may include user efficiency, clinical process and outcome metrics, adoption and utilization rates, and user satisfaction and burnout scores*.*

#### User Efficiency

This is used to quantify the number of clicks, keystrokes, mouse miles, or time spent per task. A Tier III system that democratizes efficient workflows should reduce these metrics across user populations.

#### Clinical Process and Outcome Metrics

For instance, we measure whether streamlined order sets improve prescribing accuracy, reduce redundant orders, or decrease overall encounter time. Monitoring patient throughput, wait times, or complication rates can highlight improvements in care quality.

#### Adoption and Utilization Rates

This is used to track how often recommended workflows, templates, or order sets are actually used by clinicians. High adoption signals that Tier III optimizations align with user needs and clinical realities.

#### User Satisfaction and Burnout Scores

Survey clinicians gauge whether the system’s workflow suggestions reduce frustration, documentation burden, and burnout. Positive shifts in these areas suggest that Tier III is effectively enhancing usability.

## Tier IV: External Knowledge Linkage

### Introduction to Tier IV

Tier IV proactively maintains the currency of EHR clinical content and decision support through two mechanisms. First, content may be directly linked to its derived source. For instance, registries and their integrated actions (orders, forms, laboratory or radiographic studies) could be linked to the United States Preventive Services Task Force (USPSTF) guidelines [3]. When the guidelines change, Tier IV proactively offers the configurations required to incorporate these updates. These linkages could also extend to nonclinical sources including governmental regulations or issuances, institutional policies, or payer requirements. When the Centers for Medicare and Medicaid Services update the essential elements for clinical note content, Tier IV offers the configurations to add or remove the applicable sections for efficient documentation. Second, Tier IV crawls sources of evidence and peer-reviewed literature and cross-checks these findings with existing EHR configured content. As evidence becomes available, Tier IV suggests its EHR incorporation, either as de novo content or updating existing solutions. If a trusted source of truth publishes a new clinical practice guideline, then Tier IV offers a set of EHR solutions to incorporate this clinical practice guideline across the relevant EHR workflows and positions.

### Tier IV Scenario

The USPSTF updates their breast cancer screening recommendation to begin at age 40 years versus age 50 years [3]. Because the USPSTF had been identified as a source of truth as part of Tier IV, a web-crawling, agentic AI identifies the change and suggests the requisite configuration changes to incorporate this update into the corresponding EHR patient registry. With the underlying Tier II AI system in place, changes to other associated solutions can also be performed concurrently, such as any related forms, rules, or clinical documents.

### Evaluating Tier IV

Tier IV proactively updates clinical content and decision support based on changing guidelines, regulations, and published evidence. Key metrics include update lag time, completeness of updates, accuracy of incorporated guidelines, and regulatory compliance*.*

#### Update Lag Time

This measures how quickly new guidelines or evidence-based recommendations are integrated into EHR workflows after they are published. Shorter lag times indicate that Tier IV is effectively automating the update process.

#### Completeness of Updates

This is to evaluate how comprehensively the system identifies and applies relevant updates. A high success rate suggests that the AI is accurately mapping external knowledge sources to the EHR’s configuration.

#### Accuracy of Incorporated Guidelines

This is to assess whether the recommended EHR changes align with the authoritative sources, ensuring no contradictory or partial implementations that might compromise clinical care or billing requirements.

#### Regulatory Compliance

This is to track how often Tier IV updates help ensure compliance with evolving payer, government, and institutional mandates. Fewer compliance violations or missed updates reflect a more robust external linkage mechanism.

This also helps to track how often Tier IV updates help ensure compliance with evolving payer, government, and institutional mandates. Fewer compliance violations or missed updates reflect a more robust external linkage mechanism.

## Tier V: Copilots and Assistants

### Introduction to Tier V

Finally, Tier V involves context-dependent functions that serve to enhance care efficiency, quality, and user experience for both patients and providers. These are the copilots. This tier is the current, almost exclusive focus of integrating AI within EHRs. Examples are robust, but popular ones include Microsoft’s GenAI copilot integration within Epic Systems [4] and the Nuance Dragon Ambient eXperience (DAX) AI copilot [5].

### Safeguards

Integrating AI into EHR maintenance and configuration carries inherent risks that require careful mitigation strategies. These safeguards must address proper human oversight, data security, safety testing, legal and regulatory compliance, and data standards.

#### Tiered Approval

Although AI can autonomously recommend changes to database configurations or workflows, no modifications should be pushed into production without human review and authorization (particularly for Tiers II–IV).

### Change Log and Audit Trails

We need to maintain comprehensive records of all AI-generated recommendations and subsequent human-validated changes. This includes versioning, timestamps, rationale for acceptance or rejection, and who approved the changes.

### Backout Steps

All AI-recommended changes should come with manual build and backout steps in case manual, direct human architect involvement is required to modify or rollback the change.

### Role-Based Access Controls

Authority for approving final changes should be restricted. Only designated administrators, informaticists, or clinicians with appropriate privileges should “sign off” on AI-driven recommendations [6].

### Data Security and Privacy

#### Encryption and Secure Communication

Ensure all data in transit or at rest is encrypted. For Tiers III–IV, where external data sources and registries may be accessed, secure protocols should protect patient and system information.

#### Regulatory Compliance

Any AI-based solution that handles protected health information must adhere to federal regulations and equivalent international guidelines. This includes thorough documentation of access, role-based permissions, and breach reporting mechanisms [7,8].

#### Deidentification for Training

If EHR data is used to train AI models, employ robust deidentification or anonymization methods to protect patient privacy.

### Safety Testing and Sandbox Environments

#### Staged Deployments

Deploy AI-generated configuration changes in a nonproduction environment first. Validate for unintended effects, usability impacts, and potential conflicts with existing solutions. Only after thorough testing and clinician feedback should changes move to production.

#### Automated Regression Testing

Implement continuous and automated testing routines that check clinical workflows, alert systems, and data integrity after each AI-prompted change. This helps identify errors early and prevents adverse impacts on patient care.

### Algorithm Transparency and Explainability

#### Explainable Recommendations

Tiers II–IV rely on AI to suggest or enact configuration changes. Provide clear justifications or “explanations” for each recommendation (eg, how the model determined a particular workflow optimization). Transparency bolsters trust and aids human reviewers’ decision-making [9,10].

## Ethical and Legal Considerations

### Liability and Accountability

Clearly define who bears responsibility if AI-suggested changes negatively impact patient care—whether it is the vendor, the health care institution, or a combination. Incorporate these details into institutional policies and vendor contracts.

### Regulatory Approvals

Some aspects of Tiers II–IV that meaningfully affect patient care (eg, advanced clinical decision support) may require regulatory oversight or approval from agencies. Understand and follow applicable guidelines when introducing these features [11].

## Interoperability and Third-Party Validation

### Standards-Based Implementation

Align AI-driven changes with industry standards (HL7 [Health Level Seven], FHIR [Fast Healthcare Interoperability Resources], SNOMED CT [Systematized Nomenclature of Medicine Clinical Terms], LOINC [Logical Observation Identifiers Names and Codes], etc.) to maintain interoperability.

### Independent Audits and Certification

Consider periodic third-party evaluations of AI systems and processes, focusing on data handling, software quality, and patient safety standards.

## Challenges

Although nearly every hospital and office-based physician now use a certified EHR [12], the market remains dominated by three vendors—Epic Systems (36%), Oracle Health (25%), and MEDITECH (16%) [13]. This concentration, coupled with the high up-front investment required for AI development and proprietary configuration tools, could stifle the adoption of Tiers II–IV. Overcoming this dynamic requires forging partnerships that incentivize vendors to open their proprietary configuration tools through standardized application programming interfaces and collaborative research and development initiatives. Such measures would allow third-party and in-house AI solutions to integrate, reducing reliance on vendor-specific consulting and expanding client autonomy. By adopting off-the-shelf AI modules—rather than building everything in-house or through a single vendor—small-to-mid-sized health care organizations can gradually implement Tiers II–IV at a lower cost. To make this financially viable for vendors, professional organizations, health care systems, and regulatory bodies should leverage market demand and policy incentives that reward open architectures, building upon the 21st Century Cures Act [14]. In doing so, vendors could reorient their business models—moving from fee-based solution architecture services toward engineering-focused products and support—without sacrificing profitability. This shift would ultimately accelerate innovation, lower costs, and help realize the full potential of the Elastic EHR.

## Conclusions

The adoption of the five-tiered Elastic EHR framework represents a structured approach for overcoming some of the major limitations in commercial EHR systems. By leveraging AI to manage and optimize configurations, this model addresses the inefficiencies and high costs, and downstream frustrations, associated with EHR sustainment. However, the realization of this potential faces significant hurdles, particularly due to the dominance of a few major vendors who control the necessary configuration tools and must see financial benefit in adopting such changes. Additionally, the complexity of health care environments, the need for substantial financial investment, and the lack of robust research on this topic also represent significant hurdles. Successful implementation will require collaboration, continuous research, and a balanced approach that augments medical, solution architect, and clinical informatics expertise with AI capabilities. If these challenges can be addressed, the Elastic EHR could substantially improve the efficiency, quality, and user experience of health care delivery, fully delivering on the promises of digitization within health care.

## Abbreviations

AI: artificial intelligence
EHR: electronic health record
FHIR: Fast Healthcare Interoperability Resources
HL7: Health Level Seven
LOINC: Logical Observation Identifiers Names and Codes
SNOMED CT: Systematized Nomenclature of Medicine Clinical Terms
USPSTF: United States Preventive Services Task Force

## References

[1] Craft C, Oracle Corporation (2023). White paper. Oracle Autonomous Database Technical Overview.

[2] Zhang J, Walji MF (2011). TURF: toward a unified framework of EHR usability. J Biomed Inform.

[3] (2017). U.S. preventive services task force. Published Recommendations.

[4] (2023). Modern Healthcare. Epic, Microsoft bring GPT-4 to EHRs.

[5] (2025). Nuance. DAX Copilot: Automatically Document Care and Streamline Workflows with DAX Copilot.

[6] (2016). Office of the National Coordinator for Health Information Technology (ONC). SAFER guides: safety assurance factors for EHR resilience.

[7] (2013). Department of Health & Human Services. HIPAA security rule.

[8] (2020). National Institute of Standards and Technology (NIST). SP 800-53 rev. 5: security and privacy controls for information systems and organizations.

[9] World Health Organization (WHO) (2021). Ethics and governance of artificial intelligence for health: WHO guidance.

[10] (2024). American Medical Association (AMA). Augmented Intelligence Development, Deployment, and Use in Health Care.

[11] (2019). Artificial Intelligence and Machine Learning in Software as a Medical Device.

[12] (2021). Office of the National Coordinator for Health Information Technology.

[13] Blauer T, Warburton P (2023). EHR vendor market share in the US. Becker's Hospital Review.. KLAS Research.

[14] (2016). H.R.34 - 21st century cures act. https://www.congress.gov/bill/114th-congress/house-bill/34.

